# Throwing Away the Key: The Ethics of Risk Assessment for Preventive Detention Schemes: R.G. Myers Memorial Lecture 2013

**DOI:** 10.1080/13218719.2014.893551

**Published:** 2014-03-24

**Authors:** B. McSherry

**Affiliations:** ^a^ Foundation Director, Melbourne Social Equity Institute, University of Melbourne, Australia; Adjunct Professor, Melbourne Law School and Faculty of Law, Monash University, Melbourne, Australia

**Keywords:** deprivation of liberty, ethical issues, human rights, post-sentence schemes, preventive detention, risk assessment, sex offenders

## Abstract

Preventive detention schemes that aim to protect the community from certain ‘dangerous’ individuals have long existed. While risk assessment is now pervasive in the management and treatment of many individuals, it raises particular issues when a person's liberty is at stake on the basis of what that person might do. This R.G. Myers Memorial Lecture addresses the ethical issues raised by mental health practitioners providing risk assessments for legislative schemes that involve the deprivation of liberty. It will focus in particular on Australian post-sentence preventive detention schemes for sex offenders that have been held by the United Nations Human Rights Committee to breach fundamental human rights. However, the ethical issues discussed also have repercussions for civil commitment laws that enable the detention of those with severe mental or intellectual impairments.

## Introduction

It is a great honour to be invited to deliver the R.G. Myers Memorial lecture. My introduction to the wonderful work of the Australian and New Zealand Association of Psychiatry, Psychology and Law (ANZAPPL) came about in the early 1990s when Dr Deidre Greig came to a medico-legal seminar I had helped organise at Monash University. We started chatting afterwards and, when she heard that I was interested in writing a doctoral thesis on mental state defences, she immediately handed me an ANZAPPL membership form and made me sign it on the spot. This introduction to ANZAPPL opened the door to my meeting a host of inspiring researchers and practitioners. It also sparked off my interest in interdisciplinary work, which has led me to the role I now have supporting interdisciplinary research projects dealing with social equity issues.

Dr Robert Graham Myers, better known as Bob Myers, had passed away by the time I joined ANZAPPL, but all those who spoke of him tended to do so with a smile and a twinkle in their eyes. I've heard many tales about his energy and enthusiasm, his sense of humour, the legendary dinners he hosted into the early hours of the morning, as well as his love of adventures, from trekking in the Himalayas to playing polo to deep-sea diving.

According to Professor Ian Freckelton's memoir of Dr Myers,[Fn en0001] the latter founded ANZAPPL (he was its Foundation President for almost a decade) because he was concerned that members of the three professions only interacted via the court system. He wanted to establish a forum for the exchange of ideas amongst psychiatrists, psychologists and lawyers, and this he has certainly achieved.

Dr Myers was a psychiatrist in private practice who saw giving testimony in court as an expert witness as an integral part of his work. He treated sex offenders and was concerned with counteracting some of the populist myths concerning them. As Professor Freckelton observed in this regard:

It was not a popular cause with which to be associated but when political pressure forced the government in 1990 to confront the problem of what to do with the sex offender, it was Bob who was crucial in crystallising the issues and bringing balance back to the debate.[Fn en0002]

Twenty-three years later, governments are still confronting the problem of what to do with sex offenders in the face of community outrage concentrated upon certain individuals. The reaction of five governments in Australia has been to enact post-sentence preventive detention schemes that allow sex offenders to be detained in prison or in facilities within prison boundaries for an indefinite period.[Fn en0003] These schemes rely on evidence from psychiatrists and psychologists concerning the risk of future sex offending. Recently, the New South Wales scheme has been extended to violent offenders[Fn en0004] and there is a concern that such schemes have a tendency to expand in scope.[Fn en0005]

In the tradition of Dr Myers trying to bring ‘balance back to the debate’, I want to focus on the ethics of giving risk assessment testimony for such schemes. Assessing risk is now central to many areas of civil and criminal law, and some of the issues discussed here may also apply to preventive detention schemes for those with mental and intellectual impairments.

My position is that risk assessment can be ethically justifiable in determining an individual's ‘risk needs’ for management and supervision purposes, but that it raises specific ethical problems when related to predicting future harm for the purpose of preventive detention and supervision schemes. While certain individuals should be supervised as they make the transition from prison to the community, taking away a person's liberty and ‘throwing away the key’ because of *who* they are and what they *might* do, rather than what they have done, not only breaches human rights, but focuses resources at the wrong end of the spectrum.

Let me start raising these issues by telling the story of the first man to be subjected to post-sentence preventive detention in Australia.

## The Case of Robert John Fardon[Fn en0006]

Robert John Fardon was born in 1949 in Murwillumbah, a country town in far northeastern New South Wales. His parents separated when he was very young. While it is not known what happened to his mother, his father was a labourer who served time in prison, leaving Fardon for lengthy periods in the care of an aunt and uncle on a farm.

According to Fardon, he was sexually abused by a cousin when he was seven, but when he told the adults in his family about this, he was not believed. He also later told a mental health practitioner that he had been sexually abused by other cousins and by an adult member of the family, and that he first had heterosexual sex at the age of about 11 when his father brought a woman home from the pub to have sex with him.

At the age of 12, Fardon was expelled from school after punching a teacher. He then worked on the farm or in other labouring jobs. When he was 14, Fardon left the farm after a physical fight with his father. He lived on the streets, working as an occasional farm hand and hanging out with a motorcycle gang.

When Fardon was 18, he pleaded guilty and was convicted of the attempted ‘unlawful carnal knowledge’ of a girl aged under 10, but was released on a three year good behaviour bond. Over the next decade or so, he was convicted of various offences including theft, drink driving and one count of assault occasioning bodily harm. On 8 October 1980, Fardon pleaded guilty to charges of rape and indecent dealing with a 12 year old girl and the wounding of her 15 year old sister. Fardon was 29 when he committed these offences. The sentencing judge described the rape as ‘brutal’, but he noted that because the offences were of a sexual nature, they could be described as out of character.[Fn en0007] Fardon served eight years of a 13 year sentence for these offences.

Within 20 days of being released on parole, Fardon raped, sodomised and assaulted a woman with whom he had gone to a flat to obtain heroin. On 30 June 1989, he was sentenced to 14 years’ imprisonment for those offences.

Fardon's term of imprisonment was due to expire on 29 June 2003. Two days before this date, an interim detention order was granted under the newly enacted Dangerous Prisoners (Sexual Offenders) Act 2003 (Qld). An appeal to the Queensland Court of Appeal against this order was dismissed.[Fn en0008] Fardon then appealed to the High Court of Australia which, by a majority of six judges to one, held that the Queensland Act was valid.[Fn en0009] This decision opened the door for the post-sentence preventive detention of sex offenders in Australia[Fn en0010] and similar schemes have been enacted in New South Wales, Western Australia, Victoria and the Northern Territory.

The years after the making of the first order for preventive detention were followed by a series of short-lived orders enabling supervision in the community.

Fardon was released on a supervision order in 2006.[Fn en0011] In July 2007 he was returned to custody for allegedly breaching that supervision order and stayed in prison until 19 October 2007, when he was again released on supervision.[Fn en0012] In 2008, he was charged with raping a 61 year old woman with intellectual disabilities on the Gold Coast and sentenced to 10 years imprisonment, but this was overturned on appeal.[Fn en0013] This conviction was set aside and a verdict of acquittal entered.

After that acquittal, it was then argued at a hearing for another supervision order that Fardon had breached the preceding supervision order due to his attending licensed premises with the complainant, without permission of the supervising corrective services officer, and attending the residence of an intellectually disabled person unsupervised. Despite these arguments, another supervision order was made on 20 May 2011.[Fn en0014] However, this was rescinded by the Queensland Court of Appeal.[Fn en0015]

On 13 February 2013, Justice Mullins ordered another supervision order.[Fn en0016] That decision was also overturned on appeal, and the matter was remitted for a rehearing.[Fn en0017] Once again, on 27 September 2013, Justice Peter Lyons ordered another supervision order.[Fn en0018] The Queensland Attorney-General Jarrod Bleijie was reported as stating in relation to this decision:

I've read the judgment. I've read the evidence and I form a view that this person still poses significant risk to the community … This government will do whatever is necessary to keep Robert John Fardon behind bars.[Fn en0019]

A stay on proceedings was ordered until the Attorney-General's appeal could be heard.[Fn en0020] The Criminal Law Amendment (Public Interest Declarations) Amendment Act 2013 (Qld) was then passed to enable the executive government to make a ‘public interest declaration’ in relation to a person subject to a continuing detention or supervision order such that the person could be detained in prison. A unanimous decision by the Court of Appeal of the Supreme Court of Queensland dismissed the Attorney-General's appeal against the making of a supervision order and declared the sections of the Criminal Law Amendment (Public Interest Declarations) Amendment Act 2013 (Qld), dealing with the executive government's powers, to be invalid.[Fn en0021] The Attorney-General stated that he would seek legal advice about appealing to the High Court, but, while he was on leave, the Acting Attorney-General, David Crisafulli, indicated an appeal would not proceed because ‘some of Queensland's top silks, including the Solicitor-General, all advised we were out of options’.[Fn en0022] Robert Fardon is once more on a supervision order in a building adjacent to the Wacol Prison Reserve where he ‘remains on a 24-hour curfew and cannot leave his front door without permission’.[Fn en0023]

This interplay between members of the executive and the judiciary indicates that much time and resources will continue to be spent on ‘law and order’ politics and ensuring that certain individuals are indefinitely detained. In a way, the actions of the Attorney-General to keep Fardon in prison can be seen as reflecting traditional powers for detaining insanity acquittees ‘at the governor's pleasure’. There is, in reality, nothing new in laws enabling certain groups in society to be detained on the basis of future harm.[Fn en0024] For many policy-makers, post-sentence preventive detention schemes are viewed as an essential means of protecting the public from certain groups of offenders. What is new, however, is the linking of preventive detention schemes with the notion of risk. Whether this is a step forward or not is of course open to debate.

## The Centrality of Risk Assessment to Preventive Detention Schemes

All of the Australian post-sentence preventive detention schemes for sex offenders mandate evidence of risk from mental health practitioners. For example, s 13 of the Dangerous Prisoners (Sexual Offenders) Act 2003 (Qld) requires the court to have regard to reports prepared by two psychiatrists as well as any other medical, psychiatric, psychological or other assessment relating to the offender. Section 17(4)(d) of the Crimes (High Risk Offenders) Act 2006 (NSW) requires the court to consider ‘the results of any statistical or other assessment as to the likelihood of persons with histories and characteristics similar to those of the offender committing a further relevant offence’.

In practice, mental health practitioners, in giving expert evidence for the purposes of such schemes, generally use various actuarial tools to assess the offender's risk in conjunction with clinical assessment. Some tools, such as the Static-99[Fn en0025] and the Violence Risk Appraisal Guide (VRAG),[Fn en0026] focus on variables that have been ascertained by actuarial studies as being associated with future offending. These tools are sometimes used to predict future offending and have been relied on by mental health practitioners in providing expert evidence for preventive detention schemes. Tools designed to be used in a ‘structured professional judgment’ approach[Fn en0027] such as the Historical, Clinical, Risk-20 (HCR-20)[Fn en0028] include dynamic (changeable) factors that can serve as the focus for risk *management*, but such tools are also sometimes used for risk *prediction*.

There have been a number of criticisms of risk assessment tools and their use, particularly in relation to predicting future offending as opposed to risk management. In brief, there have been criticisms of:

- *The variable-based approach*. Some researchers have argued that concentrating on trajectories of offending may provide an alternative way of exploring different types of offenders and risk profiles that are unable to be accounted for by a tool based on a variable-based approach.[Fn en0029]
- *The use of specific variables*. Many risk assessment tools do not consider current clinical variables (such as response to treatment or motivation), protective factors (such as stable employment) or variables that reduce the risk of reoffending (such as physical illness or frailty), instead concentrating on ‘static’ risk factors that cannot change over time and that cannot be changed through treatment.
- *Applying group data to the individual*. Actuarial scales are based on determining whether an individual offender has the same characteristics or risk factors as a ‘typical’ kind of offender.[Fn en0030] While an individual can be classified within a group – as ‘high risk’, ‘medium risk’ or ‘low risk’ – it is impossible to say where in this group a given person lies and therefore it is impossible to identify the *precise* risk an individual poses. In addition, the relatively low base rate for sexual recidivism means that attempting to predict who will commit further serious sexual offences will be less accurate than predicting other offences that occur at a higher rate.[Fn en0031]
- *Differences in particular groups*. The population upon which a particular tool has been validated may be quite different from the population to which it is applied. For example, many risk assessment tools were developed in Canada and so whether such tools can be appropriately applied to Australian Indigenous offenders can be questioned.
- *Applying risk assessment techniques in the courtroom*. The way in which the results of risk assessment tools are presented may lead to ‘manipulation and misinterpretation’.[Fn en0032] Mental health practitioners may not acknowledge the limitations of the tools used, leading to unnecessary detention and, in New Zealand at least, the Court of Appeal has expressed concern about the mistakes made in administering and scoring certain tools.[Fn en0033]

I have explained these criticisms in more detail in my recent book on preventive detention.[Fn en0034] What is important to note here is that the use of risk assessment tools is rarely questioned in preventive detention cases. There have been some judicial comments made about them in Australian courts, as outlined in the next section, but in general, because preventive detention legislation *requires* evidence of risk to be given by mental health practitioners, such evidence is viewed as an integral part of decisions concerning the deprivation of liberty. The ethics of this is discussed a little later. First, however, I want to briefly consider the role of law, policy and human rights in relation to these schemes.

## Law, Policy and Human Rights

As previously mentioned, the Queensland legislation was found to be constitutional by a majority of the High Court in *Fardon v Attorney-General (Qld)*.[Fn en0035] The decision in Fardon's case was limited to the issue of whether s 13 (which enables a continuing detention order to be made) of the Dangerous Prisoners (Sexual Offenders) Act 2003 (Qld) conferred jurisdiction upon the Supreme Court of Queensland which was repugnant to, or incompatible with, its integrity as a court. The majority held that s 13 of the Act was valid on the basis that the legislation dealt with a class of offender rather than an individual (distinguishing it from previous legislation that was held to be unconstitutional in *Kable v Director of Public Prosecutions (NSW)*)[Fn en0036] and because sufficient safeguards were in place to ensure the Supreme Court was exercising its judicial discretion in making preventive detention and supervision orders.

In applying the law, there is some evidence that judges prefer to make supervision orders whereby an offender is meant to be supervised in the community.[Fn en0037] However, in some instances in Western Australia, continuing detention in prison has been ordered because of a lack of suitable alternative accommodation.[Fn en0038]

In Queensland and Victoria, supervision in ‘the community’ can mean detention in separate facilities within prison grounds. In Queensland, a number of buildings adjacent to the Wacol Prison Reserve (and only 20 metres from the prison) in outer suburban Brisbane are used to house a number of offenders released on supervision orders. The buildings are surrounded by a 10 m high barbed wire fence and the outside is patrolled by a dog squad officer. This is where Robert Fardon is currently housed.

In Victoria, s 133 of the Serious Sex Offenders (Detention and Supervision) Act 2009 (Vic) enables the Governor in Council to appoint ‘any premises (including part of any building or place) other than a prison or police gaol to be a residential facility’, with the result that many sex offenders on extended supervision orders are kept at Corella Place, a 40-bed facility on de-gazetted land within the boundaries of Ararat Prison.

Some judges have made specific reference to the limitations of the risk assessment tools available in assessing whether there is an ‘unacceptable risk’ that the offender would commit a serious sex offence if not detained. In *Director of Public Prosecutions (WA) v Mangolamara*, Justice Hasluck refused to make an order for continuing detention on the basis that there was not sufficient ‘acceptable and cogent evidence’ as required under the Western Australian legislation.[Fn en0039] He rejected psychiatric evidence based on risk assessment tools because ‘the research data and methods underlying the assessment tools are assumed to be correct but this has not been established by the evidence’.[Fn en0040]

Similarly, Justice McKechnie stated in *Director of Public Prosecutions v GTR*:

While opinions based on the present predictive models may be suitable for management purposes, they lack cogency for the purposes of the DSO Act that little weight can be attributed to the results of assessments that rely on them. Accepting the view expressed that clinical interview alone is a poor predictor, it remains the case in Western Australia that as yet the tools that are being developed to increase the accuracy of predictive outcome of dangerous sexual offenders have not developed to such a stage that the evidence can be described as ‘acceptable and cogent’.[Fn en0041]

He also stated in *Director of Public Prosecutions (WA) v Comeagain*:

There remains an issue with all the predictive tools in that they have not yet been validated. They were developed, in part, to overcome the perceived and actual weaknesses of an unguided clinical assessment and have been embraced by professionals, psychiatrists and psychologists, as an improvement on an unguided assessment. Nevertheless, it would be an error to attribute a degree of scientific certainty to the tools simply because they deliver an arithmetical outcome. They remain unvalidated. Years will have to pass before a retrospective survey can determine whether and, to what extent, the predictive tools are reliable.[Fn en0042]

Despite such judicial criticism of risk assessment tools and the occasional judicial comment about how such evidence is presented,[Fn en0043] risk assessment evidence is clearly admissible.[Fn en0044] Any limitations go to the weight to be assigned to it by the judge.[Fn en0045] A petition was made to the Human Rights Committee of the United Nations under the First Optional Protocol to the International Covenant on Civil and Political Rights[Fn en0046] claiming that Robert Fardon and Kenneth Davidson Tillman's human rights had been abused under the Queensland and New South Wales legislation. In 2010, 11 of the 13 members of the United Nation's Human Rights Committee declared that the Queensland and New South Wales preventive detention schemes violated the right to liberty set out in Article 9(1) of the International Covenant on Civil and Political Rights.[Fn en0047]

The Human Rights Committee held that the onus was on the state to demonstrate that rehabilitation could not have been achieved in a manner that was less intrusive than continued imprisonment and that Australia had failed to discharge this onus. The Human Rights Committee requested that Australia provide it with information within 180 days as to the remedy taken to give effect to its views. The Australian government did not respond within this time limit. On 6 September 2011, the Australian government filed a five page document rejecting the Human Rights Committee's view that there were less restrictive means available to achieve the purposes of the New South Wales and Queensland legislation other than detention in prison.[Fn en0048]

The statement concluded with the observation that the New South Wales and Queensland governments did not consider any further action needed to be taken. Thus, despite the Human Rights Committee's finding that Fardon's and Tillman's continued imprisonment breached their right to liberty under international human rights law, the Australian government has clearly signalled that there will be no changes made to current preventive detention schemes.

## The Ethics of Risk Assessment

The Human Rights Committee's finding raises the question as to whether it is ethical to give risk assessment testimony knowing that a person's right to liberty will be taken away. There are three possible answers to this question which I want to explore. The two extreme positions are ‘always ethical’ and ‘never ethical’ with the middle ground being ‘ethical in certain circumstances’.

### Always Ethical

One extreme approach to the question of ethical risk assessment is to take the stance that mental health professionals must always perform their job and leave any ethical justifications to other professionals. Thomas Grisso and Paul Appelbaum have outlined this position as being one that leaves ‘questions of justification to the courts and society to determine, not the mental health professionals’.[Fn en0049] Thus, it is for parliament to create laws restricting the liberties of individuals and for mental health professionals to comply with what is legally required of them in this regard.

The main problem with this approach is that it makes mental health professionals agents of state control, which can have dire consequences for certain groups of individuals. One example of this occurred during the Nazi era when psychiatrists were ‘instrumental in instituting a system of identifying, notifying, transporting, and killing hundreds of thousands of mentally ill and “racially and cognitively compromised” individuals in settings ranging from centralized psychiatric hospitals to prisons and death camps’.[Fn en0050] It also ignores the obligations placed on mental health professionals through various codes of ethics to comply with ethical standards and even to seek changes in laws when they are contrary to the best interests of individuals.[Fn en0051]

### Never Ethical

The polar opposite of the ‘always ethical’ approach is to hold that it is never ethical to give risk assessment testimony. In 1984, Alan Stone started a debate in the United States about the ethics of forensic psychiatry, arguing that, as a matter of individual ethical commitment, he could not justify himself giving evidence for the purposes of the criminal justice system.[Fn en0052] While he did not go so far as to say that psychiatrists should *never* testify in court, he pointed out that those who did so risked violating the ethics relevant to those whose primary duty was to treat individuals.

Three years later, Gary Melton, John Petrila, Norman Poythress and Christopher Slobogin argued that, in relation to risk assessment, there is ‘no specialized clinical knowledge that permits categorical, or even relative conclusions about dangerousness’.[Fn en0053] As a result, they stated that mental health professionals ‘may decide that they cannot ethically offer prediction testimony’.[Fn en0054] More adamantly, Charles Ewing has argued that: ‘[t]he psychiatrist or psychologist who makes a prediction of dangerousness violates his or her ethical obligation to register judgments that rest on a scientific basis’.[Fn en0055] Similarly, Eric Janus has stated that the indeterminacy of legal terms such as ‘likely’ or ‘highly likely’ require mental health professionals to ‘make political judgments that determine the balance between public safety and individual liberty’.[Fn en0056]

The Royal College of Psychiatrists for the United Kingdom and the Republic of Ireland held a seminar on the ethics of giving evidence for extended sentencing purposes in 2002. It published an overview of the discussion in the Psychiatric Bulletin in 2005 and stated that one view aired was that ‘it is not ethically part of medicine to assist the courts in increasing punishment and public protection by applying medical skills to such a purpose’.[Fn en0057]

Refusing to engage in risk assessments for the purposes of the criminal justice system on the basis that it is unethical to do so is obviously an extreme action. Risk assessment not only informs decisions concerning the detention of offenders, but also the law relating to the civil commitment of individuals with mental illnesses, child protection and detention to prevent the spread of infectious diseases. In reality, only a few mental health professionals will take this extreme approach, but such a position does force mental health professionals to consider whether and in what circumstances giving such testimony is justifiable.

I believe that there is a middle ground between these two extreme approaches, but there remain problematic issues that need to be considered in relation to preventive detention schemes.

### Ethical in Certain Circumstances

One ‘middle ground’ approach is to extend acting for the benefit of the patient under traditional medical ethics to acting for the benefit of the offender. That is, mental health professionals should assume when accepting a call for risk assessment by the courts that there is always some prospect of treatment or benefit available for the offender concerned. This may be a less than ideal approach, however, when it is known that adequate treatment is not available.

Another approach to extending existing medical ethics is to act only where it is known that the offender will indeed receive some form of individual health benefit. Going one step further, this could mean acting only on behalf of the offender rather than for the state. This, however, assumes that mental health professionals will know when treatment is available and when it will be of benefit. It may also lead to a refusal to assess certain individuals who are not considered ‘treatable’, thus depriving them of any form of expert evidence.

A more practical approach is to use a different ethical framework for forensic psychiatry, beyond that of medical ethics, which attaches such importance to treatment. The Royal College of Psychiatrists refers to this as ‘justice ethics’, which appears to stem from Paul Appelbaum's work that forensic psychiatry should use a framework of ethics based on ‘truth’ rather than ‘beneficence’.[Fn en0058] This approach requires the fundamental value of truth telling and respect for the individual, with the ultimate goal being to advance justice rather than to act for the benefit of the individual patient. The principle of truth telling requires testifying as to what the mental health professional believes to be true, regardless of whether this advantages or disadvantages the particular offender, but it also requires an accurate presentation of the scientific data available and the consensus of the field.[Fn en0059] In relation to testimony using actuarial scales, for example, this principle would require the mental health professional to outline the limitations of the scales used.[Fn en0060]

The principle of respect for persons requires respect for the individual being assessed. In Appelbaum's words, this requires that mental health professionals should not ‘engage in deception, exploitation, or needless invasion of the privacy of the people who we examine or about whom we testify’.[Fn en0061]

In relation to risk assessment, Thomas Grisso and Paul Appelbaum have argued that a mid-way point is to see predictive testimony as neither ethical nor unethical in itself, but the *use* of the testimony may be ethical or unethical given the variability in the types of liberty restrictions involved.[Fn en0062] This may mean that the use of risk assessment to breach an individual's right to liberty is unethical and this has been argued by some psychiatrists, as discussed below.

### Unethical for Post-Sentence Preventive Detention

Danny Sullivan, Paul Mullen and Michele Pathé are three psychiatrists working in Victoria who have questioned the ethics of requiring clinicians to assess risk for the purposes of continued coercive supervision after sentence. They have pointed out that being required to give evidence for preventive detention schemes raises the spectre of mental health professionals being ‘agents of supervision, social control and monitoring’ rather than ‘independent clinicians’.[Fn en0063]

The Royal College of Psychiatrists noted that there ‘may be an ethical argument for ensuring that disagreements regarding mental disorder and risk are considered early on at the point of sentencing when the court, rather than doctors, can decide these issues’.[Fn en0064] This suggests that it is more ethically justifiable to give evidence for schemes which operate at the time of sentence than preventive detention schemes that operate *after* the expiry of an individual's sentence.

This position may run into the same problem in reality as that in relation to a complete refusal to engage in providing testimony for the criminal justice system – there will always be some mental health professionals willing to engage with legislative requirements to provide assessments of risk. However, at the very least, this position does raise the issue of mental health professionals being agents of social control and provides a starting point for distinguishing between the justifications for risk assessment for sentencing purposes within the criminal justice system as opposed to risk assessment for ‘civil’ forms of detention outside of the criminal justice system.

## Conclusion: What Would Dr Myers Have Done?

In the popular television series about commercial advertising, *The Gruen Transfer*, there is a section where the host poses the question: ‘What would Vladimir Putin or Kim Jong-un' do to get their message across?[Fn en0065] Let me adopt that to ask what would Dr Myers have done in the face of requirements to give evidence of risk for the purpose of preventive detention schemes?

In 1986, Dr Myers gave a presentation entitled ‘Responsibilities of the Forensic Psychiatrist and Psychologist’ at the annual ANZAPPL conference in which he argued for ‘a separate system of ethical guidelines and standards’ for those involved in ‘medico-legal evaluation’.[Fn en0066] He stated:

In the normal therapeutic relationship the responsibility of the therapist predominantly is to the patient whereas in the forensic area, both in civil and criminal cases, responsibility often is divided, including for example, responsibility to lawyers, the courts, the community or employers.[Fn en0067]

He also pointed out that: ‘[t]he responsibility of the therapist to protect the community can pose a troubling dilemma’ and argued that ‘there is a need for the formulation of proper guidelines’ in this regard.[Fn en0068] Although post-sentence preventive detention schemes were unimaginable in 1986, from these statements, it would seem that Dr Myers might very well be inclined towards the justice ethics approach to evidence for the courts, and would argue for clear guidelines relating to risk assessment testimony.

In Scotland, an independent body, the Risk Management Authority was established by the Criminal Justice (Scotland) Act 2003. The purpose of this authority is to set standards for, issue guidance to and accredit those involved in the assessment and minimisation of risk. I suspect Dr Myers, were he with us today, would be calling for the establishment of such a body in Australia, while also questioning why so many resources are being channelled towards keeping a small number of individuals locked away. Perhaps he would also be advocating for more resources for alternative, evidence-based means of treating sex offenders and reducing sexual offences in the community.

Marijke Malsch and Marius Duker have pointed out that detaining people in prisons or other institutions ‘works directly and speedily, which renders it attractive to policy makers’.[Fn en0069] Nevertheless, keeping individuals locked away is a costly enterprise and there is growing evidence that countries that have decreased crime the most are not in fact those that have increased incarceration the most.[Fn en0070]

Further, in interviews conducted with key personnel involved in the operation of preventive detention schemes, concerns were raised that such schemes can actually make the risk of sex offending worse for the community.[Fn en0071] As one police officer explained it, members of the community are given a false impression that they are being protected from sex offenders, thus ‘leaving the community totally at risk to the majority of offenders, who are family members and known [to] people’.[Fn en0072]

So much has changed since Dr Myers was treating sex offenders, and yet in terms of confronting the problem of ‘what to do with the sex offender’, in policy terms, perhaps the more things change, the more things stay the same. Locking sex offenders up and throwing away the key may be an instinctive response. However, in order to bring ‘balance back to the debate’, there is perhaps no better time to advocate for a focus on preventing offences occurring, targeting the causes of sex offences and assisting victims, rather than putting so many resources into keeping a small minority of sex offenders (the ones who have been caught) in prison after they have served their sentence. Hopefully, this lecture has started the ball rolling for further, more balanced and nuanced debate on this perennial problem.

## Notes

1. Ian Freckelton, ‘Bob Myers*’* (Eulogy, 24 May 1990).
2. Ibid 4.
3 These schemes have been established in New South Wales, the Northern Territory, Queensland, Victoria and Western Australia: Crimes (High Risk Offenders) Act 2006 (NSW); Serious Sex Offenders Act 2013 (NT); Dangerous Prisoners (Sexual Offenders) Act 2003 (Qld); Serious Sex Offenders (Detention and Supervision) Act 2009 (Vic); Dangerous Sexual Offenders Act 2006 (WA).
4. The Crimes (Serious Sex Offenders) Amendment Act 2013 (NSW) amended the Crimes (Serious Sex Offenders) Act 2006 (NSW) to become the Crimes (High Risk Offenders) Act 2006 (NSW) and extended the existing continuing detention and supervision schemes to ‘high risk sex offenders and high risk violent offenders’.
5. See, for example, Giorgio Agamben, *State of Exception* (Kevin Attell tr, University of Chicago Press 2005).
6. The following facts have been gleaned from a series of case reports, most of which are cited below.
7. 
*Attorney-General (Qld) v Fardon* [2003] QSC 331 [37].
8. 
*Attorney-General (Qld) v Fardon* [2003] QCA 416.
9. 
*Fardon v Attorney-General (Qld)* (2004) 223 CLR 575.
10. Bernadette McSherry, ‘Indefinite and Preventive Detention Legislation: From Caution to an Open Door’ (2005) 29 Criminal Law Journal 94.
11. 
*Attorney-General (Qld) v Fardon* [2006] QCA 512.
12. 
*Attorney-General (Qld) v Fardon* [2007] QSC 299.
13. 
*R v Fardon* [2010] QCA 317. In this case, there was evidence that Fardon had known the complainant for many years and that they had been sexual partners on other occasions. There was also evidence that the alleged offending conduct concerned a non-consensual act of digital anal penetration in the course of consensual sexual activity, which apparently ended when the complainant told him he was hurting her.
14. 
*Attorney-General (Qld) v Fardon (No 2)* [2011] QSC 128.
15. 
*Attorney-General (Qld) v Fardon* [2011] QCA 155.
16. 
*Attorney-General (Qld) v Fardon* [2013] QSC 12.
17. 
*Attorney-General (Qld) v Fardon* [2013] QCA 155.
18. 
*Attorney-General (Qld) v Fardon* [2013] QSC 264.
19. Australian Associated Press, ‘Released Serial Rapist Robert Fardon Faces Appeal’ *The Brisbane Times* (Brisbane, 30 September 2013) <www.brisbanetimes.com.au/queensland/released-serial-rapist-robert-fardon-faces-appeal-20130930-2uobz.html> accessed 28 January 2014.
20. On 4 October 2013, the judge who made the supervision order refused to stay the proceedings in *Attorney-General (Qld) v Fardon (No 2)* [2013] QSC 276, but Morrison JA granted a stay on 10 October 2013 in *Attorney-General (Qld) v Fardon* [2013] QCA 299.
21. 
*Attorney-General (Qld) v Fardon* [2013] QCA 365. The Court relied on the reasoning of the Court of Appeal's previous decision in *Attorney-General (Qld) v Lawrence* [2013] QCA 364. This was a ‘case stated’ proceeding whereby a trial judge sought the opinion of the Court as to the validity of ss 3 and 6 of the Act. The Court of Appeal found that these sections meant that the Dangerous Prisoners (Sexual Offenders) Act 2003 (Qld) would now require the Supreme Court to exercise powers repugnant to or incompatible with its function as a Court which exercises judicial power pursuant to Chapter III of the Commonwealth Constitution.
22. ABC News, ‘Sex Offender Robert Fardon to Stay Out of Jail after Qld Government Abandons High Court Appeal’ (15 January 2014) <www.abc.net.au/news/2014-01-15/qld-abandons-high-court-appeal-over-sex-offenders/5202298> accessed 28 January 2014.
23. Ibid.
24. Bernadette McSherry, *Managing Fear: The Law and Ethics of Preventive Detention and Risk Assessment* (Routledge 2014) ch 1.
25. R Karl Hanson and David Thornton, *Static 99: Improving Actuarial Risk Assessments for Sex Offenders: 2000–01* (Department of the Solicitor General of Canada 1999).
26. Vernon L Quinsey, Grant T Harris, Marnie E Rice and Catherine A Cormier, *Violent Offenders: Appraising and Managing Risk* (American Psychological Association 1998).
27. For a comprehensive overview of this approach, see Michael R Davis and James RP Ogloff, ‘Risk Assessment’ in Katarina Fritzon and Paul Wilson (eds), *Forensic Psychology and Criminology: An Australasian Perspective* (McGraw-Hill Australia 2008).
28. Christopher D Webster, Kevin S Douglas, Derek Eaves and Stephen D Hart, *HCR-20: Assessing Risk for Violence Version 2* (Simon Fraser University Mental Health, Law and Policy Institute 1997).
29. See, for example, Patrick Lussier and Garth Davies, ‘A Person-Oriented Perspective on Sexual Offenders, Offending Trajectories, and Risk of Recidivism: A New Challenge for Policymakers, Risk Assessors, and Actuarial Prediction?’ (2011) 17 Psychology, Public Policy, and Law 530.
30. Terence W Campbell, ‘Sexual Predator Evaluations and Phrenology: Considering Issues of Evidentiary Reliability’ (2000) 18 Behavioral Sciences and the Law 111; Terence W Campbell, ‘Sex Offenders and Actuarial Risk Assessments: Ethical Considerations’ (2003) 21 Behavioral Sciences and the Law 269.
31. Dominic Doyle, James Ogloff and Stuart Thomas, ‘An Analysis of Dangerous Sexual Offender Assessment Reports: Recommendations for Best Practice’ (2011) 18 Psychiatry, Psychology and Law 537.
32. Danny Sullivan, Paul Mullen and Michele Pathé, ‘Legislation in Victoria on Sexual Offenders: Issues for Health Professionals’ (2005) 183 Medical Journal of Australia 318, 319.
33. 
*R v Peta* [2007] NZCA 28, [2007] 2 NZLR 627.
34. McSherry (n 24).
35. 
*Fardon* (n 9).
36. (1996) 189 CLR 51.
37. See, for example, *Attorney-General (Qld) v Francis* [2006] QCA 324 [39].
38. See, for example, *Director of Public Prosecutions (WA) v Pindan* [2013] WASC 393; Tim Clarke, ‘WA Sex Offenders to Get State Housing’ (news.com.au, 28 October 2013) <www.news.com.au/national/breaking-news/ wa-sex-offenders-to-get-state-housing/story-e6frfku9-1226748447389> accessed 28 January 2014.
39. 
*Director of Public Prosecutions (WA) v Mangolamara* [2007] WASC 71 [2007] 169 A Crim R 379.
40. Ibid [165].
41. [2007] WASC 318 [112].
42. [2008] WASC 235 [20].
43. See, for example, *Peta* (n 33).
44. See *Woods v Director of Public Prosecutions (WA)* [2008] WASCA 188 [228].
45. See discussion in McSherry (n 24) 166–7.
46. Optional Protocol to the International Covenant on Civil and Political Rights (opened for signature 16 December 1966, entered into force 23 March 1976) 999 UNTS 171 (OP1-ICCPR).
47. 
*Fardon v Australia*, Human Rights Committee, UN Doc. CCPR/C/98/D/1629/2007, 12 April 2010; *Tillman v Australia*, Human Rights Committee, UN Doc. CCPR/C/98/D/1635/2007 12 April 2010. For an analysis of these decisions, see Ian Freckelton and Patrick Keyzer, ‘Indefinite Detention of Sex Offenders and Human Rights: The Intervention of the Human Rights Committee of the United Nations’ (2010) 17 Psychology, Psychiatry and Law 345.
48. Response of the Australian Government to the Views of the Committee in Communication No. 1635/2007 *Tillman v Australia* and Communication No. 1629/2007 *Fardon v Australia*.
49. Thomas Grisso and Paul S Appelbaum, ‘Is it Unethical to Offer Predictions of Future Violence?’ (1992) 16 Law and Human Behavior 621, 630.
50. Rael D Strous, ‘Psychiatry During the Nazi Era: Ethical Lessons for the Modern Professional’ (2007) 6 Annals of General Psychiatry 8, 8; see also Mary V Seeman, ‘Psychiatry in the Nazi Era’ (2005) 50 Canadian Journal of Psychiatry 218.
51. See, for example, American Psychiatric Association, *The Principles of Medical Ethics With Annotations Especially Applicable to Psychiatry* (s 3, American Psychiatric Association 2013) <www.psychiatry.org/practice/ethics/resources-standards > accessed 30 January 2014.
52. Alan Stone, ‘The Ethical Boundaries of Forensic Psychiatry: A View from the Ivory Tower’ (1984) 12 Bulletin of the American Academy of Psychiatry and the Law 209.
53. Gary Melton, John Petrila, Norman Poythress and Christopher Slobogin, *Psychological Evaluations for Courts: A Handbook for Mental Health Professionals and Lawyers* (Guilford Press 1987) 204.
54. Ibid 205.
55. Charles Ewing, ‘Preventive Detention and Execution: The Constitutionality of Punishing Future Crimes’ (1991) 15 Law and Human Behavior 139, 162.
56. Eric Janus, ‘Sexually Violent Predator Laws: Psychiatry in Service to a Morally Dubious Enterprise’ (2004) 364 Lancet 50, 50.
57. Royal College of Psychiatrists for the United Kingdom and the Republic of Ireland, ‘The Psychiatrist, Courts and Sentencing: The Impact of Extended Sentencing on the Ethical Framework of Forensic Psychiatry: Council Report CR 129’ (June 2004) 29 Psychiatric Bulletin 73, 75.
58. Paul Appelbaum, ‘Psychiatric Ethics in the Courtroom’ (1984) 12 Bulletin of the American Academy of Psychiatry and Law 225; Paul Appelbaum, ‘The Parable of the Forensic Psychiatrist: Ethics and the Problem of Doing Harm’ (1990) 13 International Journal of Law and Psychiatry 249; Paul Appelbaum, ‘A Theory of Ethics for Forensic Psychiatry’ (1997) 25 Journal of the American Academy of Psychiatry and the Law 233.
59. Paul Appelbaum, ‘Ethics and Forensic Psychiatry: Translating Principles into Practice’ (2008) 36 Journal of the American Academy of Psychiatry and the Law 195.
60. Stephen J Morse, ‘The Ethics of Forensic Practice: Reclaiming the Wasteland’ (2008) 36 Journal of the American Academy of Psychiatry and the Law 206.
61. Appelbaum (n 58) 197.
62. Grisso and Appelbaum (n 49) 631.
63. Sullivan, Mullen and Pathé (n 32) 320.
64. Royal College of Psychiatrists (n 57) 75.
65. The Gruen Transfer, ABC TV, Australian Broadcasting Commission.
66. Robert G Myers, ‘Responsibility of the Forensic Psychiatrist and Psychologist’ in Ellen Berah and Deidre Greig (eds), *Professional Responsibility in Psychiatry, Psychology and Law, Proceedings of the 7th Annual Congress of the Australian and New Zealand Association of Psychiatry, Psychology and Law *(ANZAPPL 1986) 93.
67. Ibid 93.
68. Ibid 96.
69. Marijke Malsch and Marius Duker, ‘Introduction’ in Marijke Malsch and Marius Duker (eds), *Incapacitation: Trends and New Perspectives* (Ashgate Publishing Ltd 2012) 1, 5.
70. Jonathan Simon, ‘Total Incapacitation: The Penal Imaginary and the Rise of an Extreme Penal Rationale in California in the 1970s’ in Marijke Malsch and Marius Duker (eds), *Incapacitation: Trends and New Perspectives* (Ashgate Publishing Ltd 2012) 15, 36.
71. Patrick Keyzer and Bernadette McSherry, ‘The Preventive Detention of “Dangerous” Sex Offenders in Australia: Perspectives at the Coalface’ (2013) 2 International Journal of Criminology and Sociology 296.
72. Ibid 298.

